# Health Care Students’ Knowledge of and Attitudes, Beliefs, and Practices Toward the French COVID-19 App: Cross-sectional Questionnaire Study

**DOI:** 10.2196/26399

**Published:** 2021-03-03

**Authors:** Ilaria Montagni, Nicolas Roussel, Rodolphe Thiébaut, Christophe Tzourio

**Affiliations:** 1 Bordeaux Population Health Research Center, U1219 Bordeaux University, INSERM Bordeaux France; 2 Inria Bordeaux University Bordeaux France; 3 Hospital Center Bordeaux University Bordeaux France

**Keywords:** contact tracing, COVID-19, mobile app, students, field survey, app, survey, monitoring, knowledge, attitude, belief, practice, communication, use

## Abstract

**Background:**

Many countries worldwide have developed mobile phone apps capable of supporting instantaneous contact tracing to control the COVID-19 pandemic. In France, a few people have downloaded and are using the StopCovid contact tracing app. Students in the health domain are of particular concern in terms of app uptake. Exploring their use and opinions about the app can inform improvements and diffusion of StopCovid among young people.

**Objective:**

The aim of this study is to investigate health care students’ knowledge of and attitudes, beliefs, and practices (KABP) toward the StopCovid app.

**Methods:**

A field survey was conducted among 318 students at the health sciences campus of the University of Bordeaux, France, between September 25 and October 16, 2020. A quota sampling method was used, and descriptive statistics and univariate analyses were performed.

**Results:**

Of the 318 respondents, 77.3% (n=246) had heard about the app, but only 11.3% (n=36) had downloaded it, and 4.7% (n=15) were still using it at the time of the survey. Among the 210 participants who had heard about the app but did not download it, the main reasons for not using the app were a belief that it was not effective given its limited diffusion (n=37, 17.6%), a lack of interest (n=37, 17.6%), and distrust in the data security and fear of being geolocated (n=33, 15.7%). Among the 72 students who had not heard of the app and were given a brief description of its functioning and confidentiality policy, 52.7% (n=38) said they would use it. Participants reported that the main solution for increasing the use of the app would be better communication about it (227/318, 71.4%).

**Conclusions:**

Even among health students, the contact tracing app was poorly used. The findings suggest that improved communication about its advantages and simplicity of use as well as clarifying false beliefs about it could help improve uptake.

## Introduction

### Background Context

Nonpharmaceutical interventions have been used to contain the spread of the SARS-CoV-2 virus [1] while effective treatments and optimal vaccine coverage are made. Besides generalized lockdown and barrier gestures, one of the solutions to limit contagion, locate clusters, and isolate them is the tracing of infected people. Contact tracing is a systematic method used as part of a disease surveillance strategy (predict, observe, and minimize) [2]. In contact tracing, an index case with confirmed infection is asked to provide information about contacted people who were at risk of acquiring infection from the index case within a given time period (between 1 week and 14 days) before the positive test result. These contacts are then alleged to be tracked, advised about their risk, quarantined, and tested [3]. Conventional or manual contact tracing is a long process demanding human resources to contact and follow up with people one by one. It can engender several delays and is potentially biased by imperfect recall of contacts [4]. These limitations can be compensated by digital contact tracing [3,5].

Several smartphone apps have been developed worldwide to automatically and rapidly trace contacts in real time. Across all continents more than 45 apps are currently used [6] and several states are planning to launch such apps [7]. The general functioning of these apps is that each mobile device running the app keeps track of other mobile devices running the app that it comes in to close contact with. When users inform their app that they tested positive, this contact log is used to determine the other mobile devices—and users—that should be notified. Existing apps use different technologies and algorithmic methods to detect contacts between mobile devices (eg, short range Bluetooth Low Energy information exchange or GPS-, WiFi-, or Bluetooth-based geolocation), to keep track of these contacts (eg, using temporary unique identifiers), to evaluate the infection risk (eg, based on a predicted distance and the duration of the contact), and to notify potentially exposed people using a centralized or decentralized network approach [8]. The effectiveness of these apps is based on the fact that individuals are systematically tested, that results of these tests are correct and communicated in the app, that the individuals who are in contact have a smartphone, and that a high proportion of smartphone users download and use the app so as to interrupt the chains of infection transmission [9].

The recrudescence of the virus after the general lockdown from March to May 2020 has especially concerned young people across France and in the Bordeaux region in particular, where incidence of COVID-19 positive cases among young adults aged 20-30 years has increased to about 252/100,000 per week (weeks 41-42) [10]. Since September 1, 2020, several hundred students of the University of Bordeaux have been tested, and 26 of them returned positive results as of November 10. Students in the health domain are on the front line in terms of contagion. First, as many students across France, they are at risk because of contact with their peers. They often meet at the university (before, during, and after classes), downtown, or for private events. During these encounters, barrier gestures and preventive measures are not always respected. Second, their role as future health-related workers might suppose that they should set the example, since they are sensitized to adopt behaviors in favor of health promotion and prevention. Third, most students in the health domain are in contact with patients directly or indirectly through their interaction with health care workers. These different situations, informal, unprotected, and in relation with potentially unknown people, are typically those in which contact tracing apps make the most sense. Furthermore, students are digitally literate [11] and are supposed to be more at ease with the downloading and use of apps. French students in the health domain are thus a priority target for the uptake of the French COVID-19–related contact tracing app.

### The StopCovid App

The contact tracing app “StopCovid” was launched by the French Government on June 2, 2020 [12]. It was developed by a team of public and private partners lead by the French National Institute for Research in Digital Science and Technology (Inria), and was available in both the Apple and Google Play stores, as it worked on iOS and Android phones. The app was based on Bluetooth signals running in the background of the phone with low-energy wireless transmission [13]. Once the app was activated, the phone logged other phones it came in to contact with, assuming these devices were running StopCovid. These logs did not include any identifying information about the user; they used random ID codes that changed every 15 minutes and were deleted once they were older than 14 days (the incubation period for COVID-19). The app did not locate the user (no GPS-, WiFi-, or Bluetooth-based geolocation); it only knew which random IDs the phone had come in to contact with. Being transparent and anonymous, the app did not collect any personal data nor contact details. If a user declared being positive for COVID-19 using a code delivered with the test results, the app would send that record of the rotating IDs to a centralized server, which in turn would send them out to other devices using the system [14]. Anyone that had the app activated who had been nearby in the last 2 weeks would be pinged with an alert. More precisely, this notification was sent if the person had spent more than 15 minutes within 1 meter of an index case. Users were then recommended to inform their general practitioner, get tested, and self-isolate, thus potentially stopping another line of transmission.

As of October 2020, StopCovid had been installed more than 2.7 million times since the beginning of June (about 4% of the French population, 67 million). Only 7969 users had declared being COVID-19 positive in the app, and only 472 notifications had been sent to potential at-risk contacts. The uptake was less than for apps in Germany (downloaded by 18 million people, about 21% of the German population, 84 million), England and Wales (16 million downloads in a population of 59 million, 27%), and Italy (9 million downloads in a population of 60 million, 26%). All these percentages are low considering that approximately 60% of the adult population would have to adopt the app to contain the pandemic [4,5]. In general, statistics show a limited use of these apps in Europe [15].

The effectiveness of the StopCovid app must be framed in the specific French context: the testing strategy was and is still unclear with limited testing capacities, tracing was not always possible, and self-quarantining was voluntary and not always followed [16]. Furthermore, three sources of risk have been identified in the StopCovid app in terms of security and data protection: (1) the hacking of the central database, (2) the reporting of fictitious or unverified cases of infection, and (3) the increased vulnerability of the smartphones themselves caused by the activation of Bluetooth [7].

### Literature Review on the Uptake of COVID-19–Related Contact Tracing Apps

Recently, several researchers have investigated the acceptability and use of contact tracing apps in the context of the COVID-19 pandemic. Some studies are based on surveys assessing the uptake of these apps among different population samples. These studies mostly refer to a hypothetical app and the intention to use it [16-24], and only a few collect information on the use of an existing tool like StopCovid [25-27]. The majority of documents reporting the real uptake of contact tracing apps are national statistics without a scientific and theoretical background. Other studies are critical viewpoints arguing on the ethical, technical, political, and scientific impact of contact tracing apps on society [2,7,9,28,29].

Concerning surveys, a multicountry cross-sectional study on 1849 adults across France, Germany, Italy, the United Kingdom, and the United States [17] showed that 74.8% of the respondents would install or keep a contact tracing app. Concerns about cybersecurity and privacy, together with a lack of trust in the government, were mentioned as the main barriers to app adoption. Another survey was conducted on 406 German adults [18], and the results showed that trust in the official app providers played an important role in the contact tracing app uptake. However, the threat appraisal of potential infection was not related to the motivation for using the app or for providing one’s own infection status to it. In Belgium [19], 48.7% of 1500 adults declared intending to use a COVID-19 tracing app. The most important predictor was the perceived benefits of the app. Respondents also reported that the clarity on how the app functioned was correlated to the will to use it. Dutch citizens were interviewed in two studies [20,23]: 41.2%-64.1% of the respondents (n=238 [20] and n=900 [23], respectively) were willing to use a contact tracing app. In one study [20], the main reason to use such an app was to control the spread of COVID-19 (30.6%). Concerns about privacy were mentioned as the main reason for not using the app (64.8%). In the other study [23], the rate of potential users strongly varied by age group: the adoption rates of the app ranged from 45.6% to 79.4% for people in the oldest (≥75 years) and youngest (15-34 years) age groups. Educational attainment, the presence of serious underlying health conditions, and the respondents’ stance on COVID-19 infection risks were also correlated with the predicted adoption rate. A national online survey on the Irish population (n=8088 responses) [21] showed that 84% of respondents would probably or definitely download the app. The most common reason for downloading the app was helping family members and friends (79%), and with a sense of responsibility to the wider community (78%). The most common reason for not downloading the app was fear that technology companies or the government might use the app technology for greater surveillance after the pandemic (41%). A longitudinal study was also conducted in Luxembourg on a representative sample of 730 adults [22]. The results showed that 72% would probably or definitely install the app if one was made available. Among motives in favor of contact tracing apps, respondents consistently mentioned responsibility toward the community and loved ones. In contrast, 11% of respondents would definitely not install the app, and their general willingness to use one was hampered by privacy and data security issues.

Acceptance of COVID-19 contact tracing apps has also been explored in France. In the first survey [27], 44% of a representative sample of 2000 French people declared that they would accept being electronically traced to avoid the spread of the virus. However, 23% were definitely against the app, and the majority of them were males and aged 25-34 years. The main reason for opposition was the fear of losing one’s freedom. Another recent survey on 1849 French adults [16] showed that the contact tracing app was rather or totally acceptable by 42.1% of the respondents. A positive correlation was found between the perceived health consequences in case of COVID-19 infection and the willingness to use the contact tracing app. Trust in the government to handle the health crisis was also strongly and positively correlated with the potential use of the app.

Concerning critical viewpoints and opinion papers, they describe the public debate on privacy concerns due to the sensitive nature of the collected data. In particular, several researchers have argued that the adoption of contact tracing apps could lead to the economic exploitation of private data and might create a mass electronic surveillance system [7]. European governments have largely debated on the use of these apps, and ethical guidelines to develop and diffuse them have also been formulated [28]. In France, researchers have particularly investigated within a theoretical framework why the population has not largely adopted the StopCovid app [2,9]. According to an opinion paper [9], there are three main reasons for the low uptake of the app: the belief that the app will not be effective because we cannot reasonably expect that its adoption rate will be sufficient to be protective, the fear of data privacy breaches due to Bluetooth and to the centralized architecture of the app, and concerns on long-term surveillance and informational privacy. According to the author, the app raises a privacy paradox [30] where immediate benefits (eg, the reduction of contacts with infected people) are preferred to the value of privacy. Since the app does not seem to be effective given its limited use, it is not worth risking the breach of one’s privacy. A second study [2] analyzed the political and scientific discourse around the promotion of the StopCovid app. Digital solutions like contact tracing apps might represent a form of alienation including government distrust. By collecting and analyzing media, scientific, and policy articles mentioning StopCovid, the study reported the contradictions of the government in handling the COVID-19 crisis based on partial and imprecise knowledge about the virus. In this context, government officials did not explain in plain language the security, privacy, data collection, processing, storage, and reuse of the StopCovid app. The app was then considered as not efficacious because of its low uptake, characterized by lack of transparency and based on alienation and coercion.

### Study Setting and Aim

This study was conducted at the beginning of the academic year at the University of Bordeaux, France, when face-to-face education was re-established, and students could freely circulate after the first general lockdown. The StopCovid app was available and downloadable for 4 months. The aim of this study is to describe knowledge, attitudes, beliefs, and practices (KABP) about the StopCovid app among students in the health domain in the Bordeaux region. The expected impact is informing on potential improvements as well as public-oriented communication strategies and appropriate political decisions to increase the app diffusion.

## Methods

### The Field Survey: Recruitment

This study was conducted within the framework of the larger ongoing i-Share (Internet-Based Students Health Research Enterprise [31]) cohort study, a French, nationwide web-based survey on the health and well-being of university students, whose principal investigators and operational staff are based at the University of Bordeaux [32].

The field survey consisted of a paper questionnaire administered face-to-face by five undergraduate students (interviewers) who had been trained to take notes, fill in the questionnaire, and describe the app to respondents. Interviewers approached their peers in the halls, canteen, courtyards, library, and study rooms at the health sciences campus of the University of Bordeaux. The collection of the data started on September 25, 2020, and ended on October 16, 2020. A sample size of 300 respondents was targeted with quotas set for the sample to be representative of the overall population of students in the health domain at the University of Bordeaux (n=16,566) in terms of sex, age (18-30 years), specific field of health-related study (medicine, dentistry, nursing, pharmacy, public health, etc), and year of study (1 to >6 years). The inclusion criteria were being older than 18 years, being a student in the health domain enrolled at the University of Bordeaux, and providing oral informed consent.

### The Questionnaire

The questionnaire was co-designed and tested by a team of 14 public health researchers and operational staff following a structured survey construction method in five steps [33]. The final questionnaire was composed of 36 items, 14 of which were common to all students (sociodemographic characteristics, suggestions for increasing the diffusion of the app, willing to recommend the app to family and friends, and fake news about data collection and sharing within the app). The other items were administered based on four different scenarios: (1) the student has already heard about the app and has downloaded it, (2) the student has already heard about the app and has not downloaded it, (3) the student has never heard about the app but would download it, and (4) the student has never heard about the app and would not download it. Specific questions were then asked depending on the scenario. Before answering further questions, students who had not heard about the app were provided a brief description of it. After responding to fake news about data collection and sharing within the app, all students were given the correct answers. Some questions were multiple choice items. The English version of the questionnaire is available in Multimedia Appendix 1. The time of administration and completion of the questionnaire was about 10 minutes. The field survey was approved by the University of Bordeaux. The oral informed consent reassured students of the anonymous format of the survey and that use of collected data was for research purposes only.

### Theoretical Framework

The questionnaire was based on the KABP scheme, which stands for the assessment of knowledge, attitudes, beliefs, and practices of populations about a specific health-related topic. This scheme is extensively used as a quantitative method (predefined questions formatted in a standardized questionnaire) that provides access to quantitative and qualitative information. Thus, items of the questionnaire were developed to capture students’ KABP about the StopCovid app. The results are discussed following the four components of this scheme.

Collected data were interpreted a posteriori through the prism of the technology acceptance model (TAM) [34] and the protection motivation theory (PTM) [35]. The TAM posits that an individual’s intent to use (ie, accept) a technology and use behavior (ie, actual use) is influenced by perceived ease of use and usefulness, which are mediated by external variables such as individual differences, system characteristics and complexity, and social influences. The TAM is especially adaptable to technology-related motivations. The PTM explains why people adopt a preventive behavior and what role fear appeals play in this process. This model comprises the threat appraisal of a potential risk (eg, infection with SARS-CoV-2) and coping appraisal of the recommended preventive behavior (eg, using StopCovid) [18]. Threat appraisal includes the perceived severity of the disease and vulnerability to it. Coping appraisal includes perceived self-efficacy (ie, belief in one’s own competence to perform a behavior even in the face of barriers) and response efficacy (ie, individuals are convinced that a behavior leads to the desired outcome and will be more likely to intend to perform the behavior). The PTM is adaptable to both health-related and technology-related motivations [18].

### Data Analysis

All data from the paper questionnaires were entered by the student interviewers in a digital database through the EpiData software version 3.1 [36]. A descriptive analysis was performed, presenting all variables and measures in the form of numbers and percentages for qualitative variables and means and SDs for quantitative variables. Chi-square or exact Fisher frequency comparison tests were used to identify statistically significant differences by age, gender, field, and year of study, modified to binary variables if necessary. Data were normally distributed. Statistical significance was defined with a *P* value <.05. Statistical powers were calculated for each frequency comparison test, chi-square or Fisher exact test, with the condition of a minimum sample size for the Fisher exact test. In calculating the power, an approximation of the normal distribution for the chi-square tests or an approximation of the Walters normal distribution for the Fisher exact tests was used. In addition, the size and proportion for each group was specified and the alpha was set at .05. The data were analyzed with SAS version 9.3 (SAS Institute).

## Results

**Figure 1:**
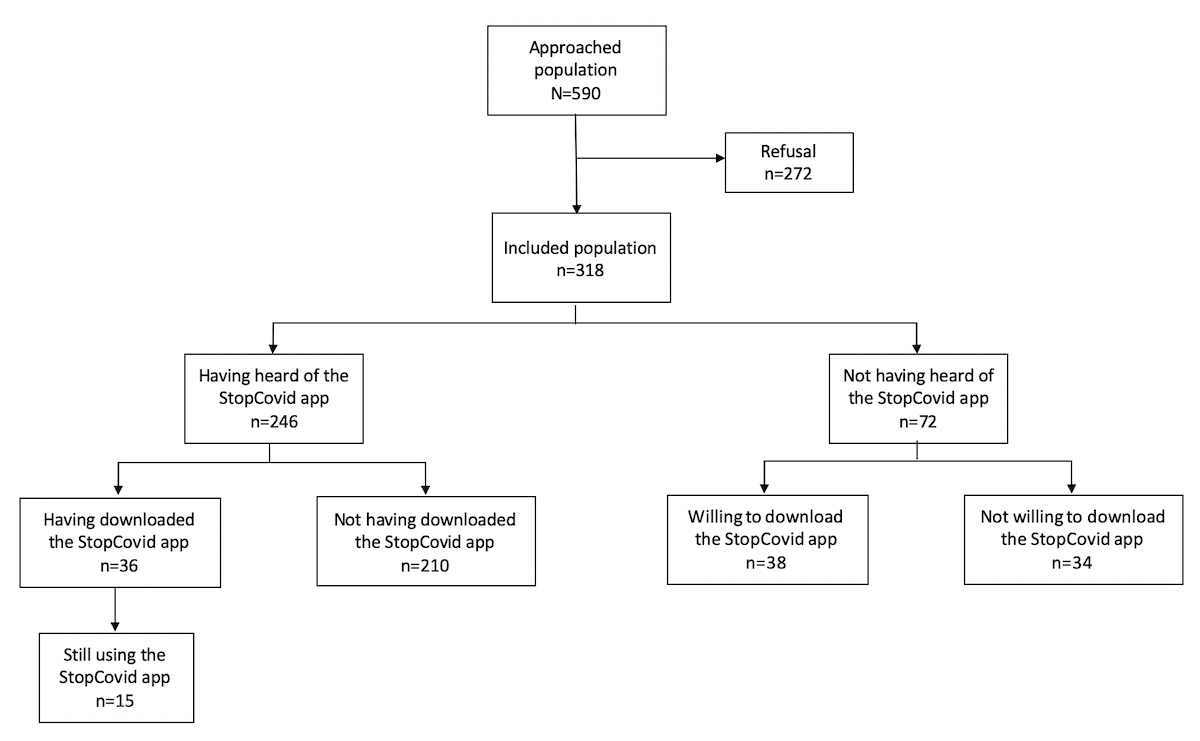
Flowchart of the study population (n=318).

A total of 590 students were approached to complete the survey after a brief explanation of its objective; 318 completed the survey, while 272 refused to participate, creating a final participation rate of 53.9%. Reasons for not participating were lack of time or no interest in the study topic. Of the 318 participants, 65.7% (n=209) were female students, and the mean age was 20.4 (SD 2.39) years. All fields and years of study were represented. The majority (n=193, 60.7%) of the participants were medical students, which is in line with the total number of medical students at the University of Bordeaux. In accordance with university-related statistics, first-year students were also the most represented (n=129, 40.6%). Figure 1 shows the flowchart of the study population, and Table 1 shows the sociodemographic characteristics with the corresponding data for the total population of health-related students at the University of Bordeaux.

**Table 1:** Sociodemographic characteristics of the study population and comparison with all students in the health domain.

Characteristics		Study population (n=318)	Total health care student population at the University of Bordeaux (n=16,566)^a^
**Sex, n (%)**			
	Female	209 (65.7)	11,713 (70.7)
	Male	109 (34.3)	4853 (29.3)
Age (years), mean (SD)		20.4 (2.39)	23.8 (—^b^)
**Year of study, n (%)**			
	1	129 (40.6)	4445 (31.2)
	2	64 (20.1)	2341 (16.4)
	3	46 (14.5)	2334 (16.4)
	4	23 (7.2)	1200 (8.4)
	5	44 (13.8)	1267 (8.9)
	>5	10 (3.1)	2651 (18.6)
	Other	2 (0.6)	—
**Field of study, n (%)**			
	Medicine	193 (60.7)	7104 (49.9)
	Pharmacy	61 (19.2)	1138 (8.0)
	Dentistry	12 (3.8)	521 (3.7)
	Nursing	14 (4.4)	3972 (27.9)
	Public health	15 (4.7)	—
	Other	23 (7.2)	1503 (10.6)

^a^Data obtained from internal university documents.

^b^Data not available.

The majority (n=246, 77.3%) of the participants had already heard about the app, mostly through the media (216/246, 87.8%) and secondly through family and friends (39/246, 15.9%). Concerning these variables, no statistically significant differences were found based on age (*P*=.09), sex (*P*=.85), field (*P*=.08), or year of study (*P*=.06).

Most of the 246 students that knew of the app correctly knew that the app was promoted by the government (n=179, 72.8%), but 25.2% (n=62) answered that they did not know who the promoter was. Male students knew significantly more than female students that the app was promoted by the government (69/85, 81.2% vs 110/161, 68.3%; *P*=.03). Female students were significantly more likely to ignore the promoter of the app compared to male students (47/161, 29.2% vs 15/85, 17.6%; *P*=.047). Students of any health-related discipline other than medicine responded significantly more than medical students that the app was promoted by a research laboratory (4/103, 3.9% vs 0/143, 0.0%; *P*=.03). Medical students were significantly more likely to ignore the promoter of the app (43/143, 30.1% vs 19/103, 18.4% ; *P*=.04). No statistically significant differences were found based on age (*P*=.06) or year of study (*P*=.17).

Among the 246 participants who had heard about the app, 14.6% (n=36) had actually downloaded it when it was first released in June 2020 (22/36, 61.1%) or with the new cases of COVID-19 at the beginning of the university year (6/36, 16.7%). Of these 36 students, 41.6% (n=15) were still using the app. Of the total 318 participants, 4.7% (n=15) of students were using the app at the moment of the survey, while those who uninstalled the app had used it from 1 day (6/36, 16.7%) to several weeks (6/36, 16.7%). The main reasons for uninstalling the app were that it was not useful (14/21, 66.7%), the respondent forgot to activate the Bluetooth (5/21, 23.8%), the app drained the phone battery (4/21, 19.0%), and too few people were using it thus making the app ineffective (4/21, 19.0%). Accordingly, students reported that the main fault of the app was that it seemed inefficient given its limited uptake (17/35, 48.6%; 1 missing). For 25.7% (9/35), the app presented technical problems like draining the battery, depending on Bluetooth, or occupying too much storage on the phone. Concerning all previous variables, no statistically significant differences were found based on age (*P* values ranging from .27 to >.99), sex (*P* values ranging from .19 to >.99), field (*P* values ranging from .13 to >.99), or year of study (*P* values ranging from .26 to >.99).

Some of these students also reported that its qualities were that it was easy to use (18/35, 51.4%) and that it was reassuring (9/35, 25.7%). Male students found the app significantly more user-friendly than female students (12/17, 70.6% vs 6/18, 33.3%; *P*=.02). Concerning this variable on the quality of the app, no statistically significant differences were found based on age (*P*=.39), field (*P*=.33), or year of study (*P*=.18).

Reasons for downloading or not downloading the app are shown in Tables 2 and 3 (multiple answers possible for each individual).

Among the 210 participants who had heard about the app but did not download it, the main reasons for not using the app were lack of interest (n=90, 42.9%), belief that it was neither effective nor useful given its limited diffusion (n=37, 17.6%), not having time to think about it (n=37, 17.6%), and distrust in data security and fear of being geolocated (n=33, 15.7%). The majority of these students might change their mind and use the app if they had more information about it through better communication strategies (n=61, 29.0%) and if more people would use it (n=54, 25.7%). Nonetheless, 26.2% (n=55) would not change their mind and would still not download the app. On the other hand, the main reasons for downloading the app were out of curiosity (13/36, 36.1%) and to protect one’s family, others, and oneself from possible infection (13/36, 36.1%).

**Table 2:** Reasons for downloading the StopCovid app.

Reasons^a^		Yes, I have downloaded the app (n=36), n (%)	Yes, I would download the app (n=38), n (%)
**Reasons for downloading the app**			
	Out of curiosity	13 (36.1)	14 (36.8)
	To protect my family, others, and myself from possible infection	13 (36.1)	24 (63.2)
	Because the government advised downloading of the app	5 (13.9)	0 (0.0)
	The app could be useful to contain the spread of the virus in general	5 (13.9)	20 (52.6)
	I am afraid of the virus, and all strategies are good to avoid it	1 (2.8)	1 (2.6)
	I was reassured the app was anonymous	N/A^b^	0 (0.0)
	Other	11 (30.6)	1 (2.6)

^a^Multiple answers possible.

^b^N/A: not applicable.

**Table 3:** Reasons for not downloading the StopCovid app.

Reasons^a^		No, I have not downloaded the app (n=210), n (%)	No, I would not download the app (n=34), n (%)
**Reasons for not downloading the app**			
	Cannot see the interest or need	90 (42.9)	17 (50.0)
	Do not like the general idea of this app	10 (4.8)	1 (2.9)
	Do not know how it works, did not get enough information on the app	23 (11.0)	2 (5.9)
	I am suspicious of this type of app	18 (8.6)	1 (2.9)
	Do not trust, because I do not know who is offering this app	11 (5.2)	1 (2.9)
	Not sure about the security of the data, fear of geolocation	33 (15.7)	1 (2.9)
	My family and friends have discouraged me from downloading it	1 (0.5)	0 (0.0)
	No storage on my phone or it is not powerful enough to have an extra app (battery, Bluetooth)	27 (12.9)	6 (17.6)
	Do not carry my phone with me at all times	2 (1.0)	4 (11.8)
	Do not use public transportation and/or do not go out much in public places (do not come into contact with strangers)	11 (5.2)	4 (11.8)
	It does not seem to be effective (too few people use it)	37 (17.6)	8 (23.5)
	Heard negative feedback on this app	7 (3.3)	1 (20.9)
	Do not really have time to think about it	37 (17.6)	3 (8.8)
	By negligence, not concerned	28 (13.3)	5 (14.7)
	Not sure what it is all about, the principle and/or the functioning	11 (5.2)	N/A^b^
	Other	15 (7.1)	2 (5.9)

^a^Multiple answers possible.

^b^N/A: not applicable.

The 72 students who had never heard about the app were asked to imagine its content and objective: 41.7% (n=30) reported that it was an app providing advice and information about COVID-19, 29.2% (n=21) reported that it was an app to limit the spread of the virus, 29.2% (n=21) did not know, and 15.3% (n=11) answered “other.” After a short description of the app, 52.7% (n=38) said they would download it. The reasons for downloading or not downloading the app are similar to those provided by the sample who had heard about the app. Among the 34 students who had never heard about the app and were still not willing to download it after a brief description, 32.4% (n=11) would not change their mind, 17.6% (n=6) would download it if more people used it, and 11.8% (n=4) would download it if they had a better mobile phone.

Concerning the functioning of the app, 83.3% (30/36) of the respondents said that they were able to explain it. However, when further asked about geolocation, access to contact information, and how data were transmitted and stocked, their answers were mostly incorrect. As expected, students who had not heard about the app before, but who were presented a quick description of it during the survey, provided correct answers more than their peers. Detailed results are shown in Table 4.

**Table 4:** Knowledge and beliefs about the functioning and data management of the StopCovid app.

Knowledge and beliefs		Yes, I have heard about the app (n=246), n (%)	No, I have not heard about the app (n=72), n (%)	Total (N=318), n (%)
**StopCovid geolocates you and tracks your movements**				
	No (correct answer)	78 (31.7)	44 (61.1)	122 (38.4)
	Yes	138 (56.1)	19 (26.4)	157 (49.4)
	Not sure	30 (12.2)	9 (12.5)	39 (12.3)
**StopCovid collects your contacts and knows their names (on the phone, on social networks, etc)**				
	No (correct answer)	178 (72.4)	56 (77.8)	234 (73.6)
	Yes	37 (15.0)	5 (6.9)	42 (13.2)
	Not sure	31 (12.6)	11 (15.3)	42 (13.2)
**StopCovid detects people around you and knows their names (physical contacts)**				
	No (correct answer)	124 (50.4)	45 (62.5)	169 (53.1)
	Yes	82 (33.3)	15 (20.8)	97 (30.5)
	Not sure	40 (16.3)	12 (16.7)	52 (16.4)
**StopCovid has access to your personal data and communicates them**				
	No (correct answer)	191 (77.6)	55 (76.4)	246 (77.4)
	Yes	28 (11.4)	5 (6.9)	33 (10.4)
	Not sure	27 (11.0)	12 (16.7)	39 (12.3)

Finally, all 318 participants were asked about factors for increasing the use of the app. For the majority (n=227, 71.4%), the solution was a better communication strategy. Other factors were making the app compulsory (n=45, 14.2%), registering more COVID-19 cases (n=30, 9.4%), more information and explanations about the app (n=21, 6.6%), better technical features (n=10, 3.1%), and “other” (n=64, 20.1%).

## Discussion

### Principal Findings and Interpretation

As far as knowledge is concerned, 1 out of 5 students had never heard about the StopCovid app; this rate is surprisingly high considering that students in the health domain should be informed of existing tools to limit the spread of COVID-19. Those who knew the app had heard about it mostly through the media (216/246, 87.8%). The majority of students (179/246, 72.8%) correctly knew that the app was promoted by the government. However, concerning the functioning of the app, some students did not know how the contact tracing system worked and how data was managed: percentages of errors in describing the app ranged from 10.4% (33/318) to 49.4% (157/318). For them, the app was not straightforward; in the light of the TAM, reduced ease of app use determines lower acceptance. Furthermore, as an external variable, system complexity might have mediated the perception of ease of use for the app. In general, limited information about a tool, from knowing that it exists to knowing how it works, is associated with poor use of the tool itself. Consistently, when asked how they would improve StopCovid adoption, 71.4% (227/318) of students suggested deploying better communication and information strategies for increasing knowledge about the app.

In terms of attitudes, students reported several reasons for not downloading or uninstalling the app. Their intention not to use the app was mostly due to the fact that they considered the app as neither useful nor effective (14/19, 73.7%), especially because few people were using it. As suggested by the TAM, perceived usefulness is a key determinant of acceptance of a new technology, which justifies the low adoption of StopCovid by students of our sample. Technical issues like draining the battery, use of Bluetooth, and mobile phone storage were also mentioned (9/19, 31.0%). Once again, according to the TAM, technological components are strictly related to acceptance of a digital tool. On the other hand, reasons to download the app included wanting to protect one’s family and friends: percentages ranging from 36.1% (13/36) to 63.2% (24/38). According to the PMT, the effort or cost (ie, response efficacy) of using the app was worth it to protect others from the virus. A few students (5/36, 13.9%) reported that the promotion of the app by the government motivated them to use it. This result might reflect a certain degree of confidence in political authorities.

As for beliefs, when specifically asked about the functioning and data management of the app, half of the total sample believed that the app was intrusive: it could geolocate them, track their movements, and access phone contacts. For 1 out of 6 students, fear of being tracked and that data could be collected and shared discouraged them from downloading the app. This false belief might have been nourished by the fact that students could have heard in the media that data breaches were possible, directly on their phones through Bluetooth, and that central servers could be violated. According to the PMT model, if beliefs do not support the recommended preventive behavior, probability of adopting such behavior is reduced. Furthermore, if a data breach is felt like a threat, individuals would be motivated not to download the app to protect themselves. Among those who received a clear explanation of the functioning of the app and its confidentiality policy (no geolocation and no access to personal data), 1 out of 2 students felt reassured and would finally download the app. These results confirm that beliefs, either true or false, influence behavioral intention.

Finally, concerning practices, 14.6% (36/246) of participants had actually downloaded the app, and in the whole sample, only about 4.7% (15/318) were still using it at the time of the survey, which is in line with national statistics concerning the general French population (4%). Furthermore, 26.2% (55/210) of respondents would not change their mind and still would not use the app. Possible justifications could include the fact that young people might perceive the pandemic as not dangerous for them. Epidemiological data confirm that COVID-19 is fatal mostly for people older than 60 years or who have a chronic disease [37]. Within the PMT framework, considering the illness as not too severe and perceiving vulnerability as low are related to the limited need to adopt a specific health-related behavior, which corresponds, in our case, to the downloading of the app. Along the same line, students might feel their competences (ie, self-efficacy), such as barrier gestures, are enough to prevent the virus, independent of app use. These are potential explanations for the general lack of interest in the app showed by our sample (90/210, 42.9%).

### Comparison With Prior Work

Overall, international and French surveys (eg, [16-18,27]) have showed a higher acceptance of a contact tracing app than the real use we found in our study. Percentages of potential use of the app range from 38.4% [16] to 84% [21], which are substantially higher than the 4.7% (15/138) of respondents in our study who were using StopCovid. However, the lowest rates of acceptance for a contact tracing app were found mostly in France: 38.4% [16] and 44% [27]. Inversely, in our sample, 26.2% (55/210; having heard about the app) to 32.4% (11/34; not having heard about the app) of students would not change their mind and would not use the app at all. This percentage is higher than in the Luxembourg survey (11%) [22] and the Irish survey (7%) [21], but similar to one of the French surveys (23%) [27] and the Belgian survey (20.4%) [19]. Discrepancies between our study and previous surveys might be explained by the fact that the latter asked hypothetical questions about future behavior; high levels of intended installations might not directly translate into actual installation. It might be harder for respondents to visualize how such apps work, thus limiting the reliability of their responses compared to a real-life scenario. Furthermore, optimistic results found in previous surveys might be due to the fact that they had been conducted when the epidemic was on the rise and before digital contact tracing had been widely discussed in the media, especially in relation to data security. This might be the case especially for France [16]; citizens’ opinions might have changed when the StopCovid app was developed and controversies about it were raised in public debate in Spring 2020.

A study exploring the real uptake of an existing app in Singapore, the TraceTogether app, had an uptake of 20% [25]. This higher percentage, compared to our study, might be justified by the fact that Asian countries are often referred to for their decisive and authoritative responses to pandemics. More convincing communication around the app might have increased its uptake. Furthermore, TraceTogether has been a real pioneer in COVID-19–related apps given its high performance, which might have further supported its use. However, the TraceTogether app received criticism for draining mobile phone batteries, which was one of the faults reported in this study about the StopCovid app. In fact, excessive use of battery and data storage were mentioned by some of our students as reasons for not downloading the app (27/210, 12.9%), and 25% (9/36) had uninstalled the app because of these technical problems.

In our study, the three main reasons for not downloading the app were lack of interest (90/210, 42.9%), belief that it was neither effective nor useful given its limited diffusion (37/210, 17.6%), and not having time to think about it (37/210, 17.6%). No previous survey has reported these same reasons, even if in the literature the notion of contact tracing apps’ effectiveness has been widely discussed [5]. Students were aware that if the app is not used by a consistent number of people, it is not efficacious at all. In general, our sample expressed disinterest in the app. The reasons should be further explored, but we might suppose that the app was not considered as useful given the other restrictive measures in place: national lockdown, social distancing, and barrier gestures.

Distrust in data security and fear of being geolocated were mentioned by our sample as the fourth reason for not downloading the app (33/210, 15.7%). Researchers worldwide, from Europe to Asia, have emphasized the privacy controversies of contact tracing apps, presenting them as the main fault of this type of technology [9,17,22,25,38]. Fears of greater surveillance and that the app might be hacked are mentioned in these studies as barriers to app use. In France, the question of data privacy related to the StopCovid app has been particularly explored; the app does not come without short-term and long-term risks of privacy and surveillance. French people face a moral dilemma: the app can prevent the spread of the pandemic, especially protecting older adults, but limit freedom to move, data security, and privacy, which are usually sensitive issues in the French culture and politics [9]. However, this did not seem to be a source of much concern in our study compared to perceived ineffectiveness and inutility of the app. For our sample, uptake of the app might not necessarily be a matter of data security or trust in the government but a question of practicality and usefulness. This result might be explained by the fact that the youth are already used to sharing their information online (eg, in social networks) and are not as concerned by cybersecurity [9]. In line with this, none of our respondents mentioned being reassured that it was anonymous as a reason to download the app. Furthermore, the proportion of students who received an explanation of the functioning of the app were comforted about the fact that no private data was collected, users were not geolocated, the app did not access contacts, and that only an anonymous code was transmitted to a centralized server by Bluetooth and deleted after 14 days. For the 15.7% (33/210) of students who were cautious about data security, following the PMT model, severity of and vulnerability to data misuse might have reduced their motivation to use the app, as reported in the German survey [18]. In any case, information should be more accurate on data security since this issue could discourage young people from downloading the app. Exact data management in the contact tracing app needs to be clarified to guarantee the respect of the user’s privacy.

Similarly, trust in the authorities was mentioned in previous research as a factor influencing the uptake of the app: individuals who have less trust in their national government were also less supportive [17]. Despite not exploring the notion of trust in the government, we observed that 13.9% (5/36) of those who downloaded the app were motivated from the advice by the government. This response option might be considered as a proxy for trust in the government. Although data from a larger sample is needed to corroborate this result, we might assume that the political discourse has an impact on the diffusion of the app, whether positive or negative.

The main reasons for downloading the app were curiosity (13/36, 36.1% of those who downloaded the app and 14/38, 36.8% of those who would download the app) and wanting to protect family, others, and oneself from possible infection (13/36, 36.1% and 24/38, 63.2%, respectively). The second reason was also reported in the Luxembourg survey [22] and in the Irish study [21]. Students who had received an explanation of the functioning of the app reported twice as much as the other students the fact that the app could prevent them and their beloved ones from the spread of the virus. Similar to half of the Luxembourg study’s sample [22], for some students, a good reason for installing the app was that it may stop the epidemic: percentages ranging from 13.9% (5/36) for those who downloaded the app to 52.6% (20/38) for those who would download the app. In general, when provided with a clear explanation of the app, 1 out of 2 students was convinced to download the app because of our study. In this line, 10.9% (23/210; of those who had heard about the app) and 5.9% (2/34; of those who had not heard about the app) of our respondents said that they would not download the app because they did not have enough information on how it worked. Similarly, participants in the survey conducted in Belgium [19] declared that lack of clarity on its functioning was among the reasons for not downloading the app. This suggests that providing clear information on the objectives of the app might promote its uptake.

Finally, we must consider the specificity of our population compared to previous research. Although studies were conducted on the general population (mostly nationally representative samples), we presented data exclusively from university students in the health domain. Our sample might have felt less concerned by the pandemic or simply less interested in this type of digital solution, or studying in the health domain and potentially working in hospitals might have made a contact tracing app for our respondents superfluous since they could be in contact with patients who were infected. A qualitative study would be useful to analyze the motivation for not using the app in this specific population.

### Strengths and Limitations

This was one of the first studies reporting data on students’ KABP about a contact tracing app in a pandemic context. Previous studies have explored the intention of downloading this type of app as a general idea but were not based on a developed and currently diffused app [17-19,23]. Reasons for downloading and using the app were presented to inform future steps to increase its diffusion. The specific focus on students was another strong point of this study: young people were especially concerned by the transmission of the virus in subsequent COVID-19 waves. Mobilizing this population to adopt the app is pivotal in this particular epidemiological context.

Limitations of this study include the relatively small sample. More than 300 students in the health domain were interviewed among a total population of 18,000 students. Findings cannot be generalized, but the sample was recruited according to quota sampling to be, as much as possible, representative of sex, age, specific field of study (from medicine to pharmacy), and year of study. This might increase the representativeness of the interviewed population group. However, it is possible that students interested in the topic were more willing to participate so that the final sample might be biased (self-selection bias). The small sample also justifies the few significant differences that were identified. This is confirmed by the low statistical powers that were obtained following performed statistical tests (<0.50).

### The New Version of the StopCovid App: TousAntiCovid

StopCovid received several criticisms and even the French Prime Minister Jean Castex officially declared not having downloaded the app. The government considered the low uptake of the app as the main issue of StopCovid. Some weeks after the implementation of this study, on October 22, 2020, the French President Emmanuel Macron announced the launch of a new contact tracing app, TousAntiCovid. There are two main differences between the two versions of the app: embedded functionalities and promoting strategy. Concerning functionalities, they include provision of information on new cases (effective R, incidence rate, hospitalizations, etc), advice and news about COVID-19, geolocation of testing centers, and generation of the mandatory certificates for permission to be outdoors during the lockdown. This last functionality, in particular, might have increased the download and use of the app. As for promoting strategy, it was more intense but less coercive and more transparent about the app compared to StopCovid. The French President and Prime Minister were strongly engaged in the communication campaign from the beginning, whereas StopCovid had mainly been promoted by the Minister of Health and by the Digital Secretary of State [2], who have less influence on the general population. In light of this, a new survey on the TousAntiCovid app might provide different results to compare with our study.

### Implications

This survey was conducted as the preliminary phase of a complex intervention aimed at promoting the uptake of the StopCovid app among students in the health domain at the University of Bordeaux. After this first appraisal of KABP about StopCovid, the next steps are to implement a series of actions at the university. Professors and lecturers have been mobilized and trained to present the contact tracing app to their students during classes. Furthermore, students will also be informed by more communication such as short videos on the university website and intranet, flyers, posts on social networks, and posters. Student ambassadors and associations will also be involved in the diffusion of the app. This complex intervention will be evaluated through a second series of random field surveys aimed at observing an increase in the number of app downloads. Depending on the results of the evaluation, the intervention will be extended to students in other fields of study at the University of Bordeaux and other universities across France.

### Conclusion

Overall, we found broad support for app-based contact tracing, notwithstanding the low uptake of StopCovid among French students in the health domain. The results suggest that the functioning and purpose of the app were not well known and appraised among participants, especially because of the lack of factual communication. Efforts are to be taken in these terms to increase knowledge about the new TousAntiCovid app, diffuse its adoption, and consequently improve preventive behaviors among young people who represent an important target audience in the strategies to limit the transmission of COVID-19. The way the app traces contacts should be better explained so as to maximize its download and consequential use by eliminating any potential false belief. The French government should be particularly involved in providing quality, clear, appropriate, and straightforward information about TousAntiCovid.

## Appendix

Multimedia Appendix 1 English version of the StopCovid field survey questionnaire.

## Abbreviations

i-Share: Internet-Based Students Health Research Enterprise
KABP: knowledge, attitudes, beliefs, and practices
PTM: protection motivation theory
TAM: technology acceptance model
